# What Every Girl Ought to Learn

**Published:** 1887-11

**Authors:** 


### WHAT EVERY GIRL OUGHT TO LEARN.

We cannot too highly commend the following ingenious summary of what every girl should learn, whatever her social status, condition or future expectations, which we cut from the *Secular Review*. The writer thoroughly understood his subject, and every girl of good common sense should understand and profit by it.

She should learn to use her senses to the best advantage, especially her hands and eyes—in other words, she should have an "education by doing."

She should learn how to sew, darn and mend.

She should learn to cultivate flowers, and to keep the kitchen garden.

She should learn to make the neatest room in the house.

She should learn to have nothing to do with intemperate or dissolute young men.

She should learn that tight lacing is uncomely, as well as injurious to health.

She should learn to regard their morals and habits, and not their money, in selecting her associates.

She should learn that twenty shillings make a pound.

She should learn how to arrange the parlor and library.

She should learn that there is nothing more conducive to happiness than a comfortable house dress. The idea that anything is good enough about the house and in the kitchen is a very grave mistake.

She should learn to observe the old rule, "A place for everything and everything in its place."

She should learn that music, drawing, and painting are real accomplishments in the home, and are not to be neglected if there be time and money for their use.

She should learn the important truism, "That the more she lives within her income the more she will save, and the farther she will get away from the poorhouse."

She should learn that a good, steady, thoughtful mechanic, farmer, clerk, or teacher, without a shilling, is worth more than forty loafers or nonproducers in broadcloth.

She should learn to embrace every opportunity for reading, and to select such books as will give her the most useful and practical information, in order to make the best progress in earlier as well as late home and school life.

She should learn that a plain, short dress, comfortably made, is a very regiment of strength ; and wash goods are decidedly preferable, because, with a clean dress, even if it be only a cheap print of home-spun a woman puts on a kind of beauty, and there is something in clean clothes marvellously helpful to being clean-tempered.

She should learn how to manage a house. Whether she marry or whether she do not, the knowledge will almost certainly be of service, and at some time of her life will probably be a necessity to her.

"A girl, whether rich or poor, whose education has been conducted upon a plane so high that to become a fashionable idler or an inconsequent gossip or dawdler would be impossible, will be the one most earnest in considering the holy purposes and in fitting herself for the responsibilities of the most serious step of her life—marriage."

